# Validation of the Scale on Parental Feeding Behaviors (ECOPAL) for Caregivers of Mexican Children

**DOI:** 10.3390/nu15173698

**Published:** 2023-08-24

**Authors:** Marina Liliana González-Torres, Xochitl Garza-Olivares, Gabriela Navarro-Contreras, Lidia Alejandra González-Orozco

**Affiliations:** 1 Department of Psychology, Autonomous University of Aguascalientes, Aguascalientes 20100, Mexico; lidia.gonzalez@edu.uaa.mx; 2 Department of Clinical and Health Psychology, Tecnológico de Monterrey, Monterrey 64849, Mexico; xochitl.garza@tec.mx; 3 Department of Psychology, University of Guanajuato, Guanajuato 37670, Mexico; g.navarro@ugto.mx

**Keywords:** parental feeding behaviors, healthy feeding, prevention, childhood obesity, upbringing

## Abstract

Parental feeding practices can be crucial to preventing childhood obesity. This study aimed to validate a self-applicable instrument for evaluating the diverse parental feeding behaviors of Mexican caregivers based on the theoretical constructs of coercive control, structure, and autonomy support. The scale’s content validity achieved significant values when assessed by expert judges, with moderate intensity in congruence (Kendall’s W = 0.462; *p =* 0.000) and clarity (Kendall’s W = 0.369; *p =* 0.001). The participants were 1185 Mexican adults (32.7 ± 7.6 years of age, 97% women, and 90% mothers) responsible for the main meal of at least one child (4.8 ± 3 years old). The data were subdivided randomly for an exploratory factor analysis (*n* = 581) and a confirmatory factorial analysis (n = 604). The first analysis grouped the items into 11 factors, with an accumulated variance of 63.9%. In the confirmatory analysis, a 10-factor model showed a better fit (CMIN = 1531.5, *p* < 0.001, CMIN/df = 2.20, RSEA = 0.045, CFI = 0.92, TLI, 0.91, and NFI = 0.87). The factors in this model were (1) the disposition of non-recommended foods, (2) nutritional education, (3) pressure to eat, (4) praise for healthy eating, (5) monitoring of consumption, (6) structured offer of fruits and vegetables, (7) consumption conditioning, (8) overt restriction, (9) guided choices, and (10) covert restriction. The Cronbach’s alpha value was 0.816. Therefore, this scale presents good psychometric properties with which to evaluate the frequency of child caregivers’ feeding behaviors in the context of ten different feeding practices in Mexico’s urban areas and contributes to the knowledge of current practices in the Mexican population. It also evaluates changes resulting from future interventions that promote eating practices that favor the formation of healthy eating habits.

## 1. Introduction

Globally, at least one in three children under the age of five is malnourished or overweight [1]. In Mexico, both problems are present. However, childhood obesity has the most significant impact, as Mexico is considered a country with one of the highest worldwide rates of obesity [2], which has aroused a growing interest in the study of obesity. It has been noted that a fundamental step in the search for a means of preventing childhood obesity is the analysis of parental feeding practices [3,4], which are a set of behaviors and actions that parents carry out to influence the eating behavior of their children [5]. This definition can be applied to many behaviors carried out by parents, but there needs to be more clarity about its operationalization and its consequences on child eating behavior [6].

The results of some studies suggest practices that can be beneficial for the development of healthy eating habits by promoting autonomy, stimulating self-regulation and self-control, such as providing nutritional education, involving children in the selection of foods, diet, motivation, modeling, reasoning, and negotiation, among others [5,6,7]. However, it has been found that parents receive little guidance on how to contribute to the development of their children’s feeding autonomy from an early age and how to manage the problems of feeding children, for example, knowing how to face a refusal to consume food [8].

The family context determines lifestyle development, including activity and eating patterns [3]. Recently, an observational study of Mexican caregiver–child interactions in a natural feeding context, in which parental feeding behaviors were operationalized from previous theoretical proposals [5,6], revealed that the adults offered low proportions of fruits and vegetables to the children during their meals, and that eating together with the child, praising the child’s intake, and highlighting the properties of foods were highly probable behaviors to appear with the acceptance of food. Still, these behaviors were infrequent in the caregivers [9]. However, this observational study evaluated a few parents (n = 10), and evaluating a larger sample would be very costly. A questionnaire for assessing family eating practices is a valuable research tool for nutritionists, psychologists, nurses, and other specialists seeking to understand and promote healthy eating habits in children [10].

Current measures only assess select parental feeding practices and conceptualize these practices differently [10], and fewer culturally appropriate instruments exist for the Latina population. In Mexico, two instruments have been adapted for the study of parental feeding practices, both with good psychometric properties. The Child Feeding Questionnaire [11], adapted and validated for Mexico [12], presents a Cronbach’s alpha of 0.858, which was validated in a sample of mothers of children between 5 and 12 years of age, and the Comprehensive Feeding Practices Questionnaire [13], adapted for Mexico [14], obtained a Cronbach’s alpha > 0.60; its validation process also occurred in a sample of mothers, but they had preschool-age children. In addition, these adaptations only consider one of each child’s caregivers: the mother. These questionnaires have not been applied to other caregivers such as grandmothers, parents, or other individuals responsible for feeding children.

Although there are instruments for measuring parental feeding practices that have been validated in the Mexican population, these include items that do not directly reference behaviors but rather attitudes and beliefs about infant feeding. For example, the Child Feeding Questionnaire [11,12] presents item 30, which says, “If I did not guide or regulate their feeding, my child would eat less than he/she should”, and item 28, “I have to be especially careful to make sure my daughter eats enough”, both of which refer to beliefs and not necessarily to actions. Similarly, the Comprehensive Feeding Practices Questionnaire [13,14] presents items such as item 46, which indicates, “I try to eat healthy foods in front of my child even though I will not be my favorites”, and item 47, “I try to show enthusiasm about healthy foods”, which refer to behaviors, but lack precision when evaluating the attempt and not the performance of the behavior. In the study of parental eating practices, it is imperative to establish which behaviors are carried out and at what frequency.

For its part, the HomeSTEAD survey [10], which consists of three subscales and 86 items, assesses 24 parental feeding practices based on the authors’ proposed theoretical classification [5], i.e., coercive control, structure, and autonomy support practices; the latter two represent positive constructs that promote healthy eating behaviors in children. Each of the subscales of the instrument proposed by the authors shows an acceptable internal consistency (Cronbach’s alpha > 0.62). However, the sample with which this instrument was validated comprised inhabitants of the United States of America. Recently, a Portuguese version of the HomeSTEAD family food practices survey was validated in a sample of parents of children aged 3–12 years old and proved an acceptable level of internal consistency (Cronbach’s alpha > 0.61) [15]. At present, the survey has undergone no validation in the Spanish-speaking population.

In addition, several items in this survey have the same lack of precision with respect to specific behaviors; for instance, “My child learns to eat healthy snacks from me” is an affirmation that does not describe how the parent achieves the result. The following is another example: “How often do you plan your family’s meals to provide a variety of food groups?” This question does not specifical ask how often the participant includes a variety of food groups in their family’s meals.

Therefore, this work aimed to develop and validate a self-applicable instrument that evaluates the frequency with which caregivers of children between the ages of 1 and 11 perform parental eating behaviors that can describe parental feeding practices according to the classification in Table 1 [5]. The items developed in this instrument are not a translation of the HomeSTEAD survey, since they were written with a focus on defining the constructs based on Mexican culture, and care was taken to ensure that each item refers to specific, observable behaviors.

## 2. Materials and Methods

**Figure 1:**
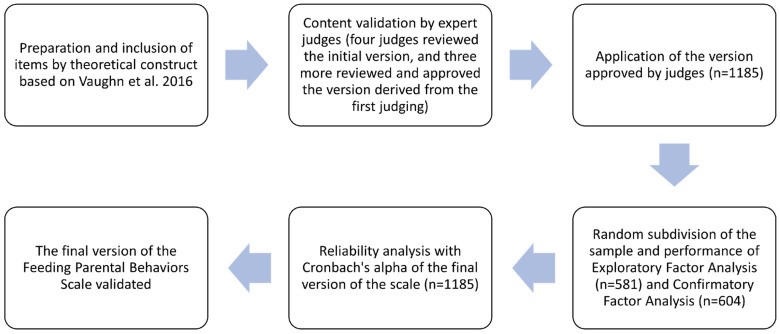
Diagram of the process of validating the Scale of Parental Feeding Behaviors.

The scale construction and validation process followed the steps shown in the diagram in Figure 1.

### 2.1. The Development of the Scale on Parental Feeding Behavior

The objective of this instrument is to evaluate the weekly frequency with which caregivers utilize behavioral strategies when feeding children between 1 and 11 years of age. The items were developed by three experts (bilingual psychologists), considering the constructs [5] shown in Table 1. The operational definitions allowed the constructs to be included as instrument dimensions.

### 2.2. Content Validation by Expert Judges

The content validation method [16] provided an instrument for measuring the judges’ verdicts on the items and the stages in the content validation process.

The experts were selected according to their knowledge and experience concerning parental feeding practices (two psychologists), psychometry (one psychologist), or both (two psychologists and one nurse). All are postgraduates in their fields (five Ph.D. degrees and one Master’s degree). The six expert judges are from different states in the country’s north, center, and south (Aguascalientes, Guanajuato, Jalisco, Nuevo León, and Veracruz).

In the first stage, four judges evaluated each item of the first version of the instrument in terms of its congruence (whether the item has a logical relationship with the dimension or indicator that measures) and clarity (whether the item is easily understood; if its syntax and semantics are adequate) on a scale from 1 to 4, according to a widely used instrument [16]. The mode and median of each item were obtained for the judges’ evaluations, and the agreement between judges was calculated via Kendall’s W to validate the content of the congruence criteria (Kendall’s W = 0.462, X2= 118.24, and *p =* 0.000) and clarity (Kendall’s W = 0.369, X2 = 94.518, and *p* = 0.008. Likewise, the judges were asked to provide comments, observations, or suggestions for each item’s correction. Items that obtained a median score equal to or less than 3.5 were corrected, considering all of the judges’ observations. In the second stage, the updated version was sent to three final expert judges (psychologist researchers with Ph.D. degrees in parental feeding practices and with experience in psychometry) who agreed to approve the final version, which contained 80 items.

The cultural adequacy of the instrument was assessed in a field test, as recommended by the literature [17], with 13 Mexican mothers of children 1–5 years of age. These participants indicated that the items were clear and understandable.

### 2.3. The Application and Validation of the Instrument

#### 2.3.1. Participants

The participants comprised 1185 Mexican adults responsible for the main meal of at least one child in Mexico’s urban zone. The participants were identified through non-probabilistic sampling via the initial dissemination of the publication in an essential educational institution, in addition to its promotion on social networks in November 2022, taking into account the following considerations about the public to which it was directed: men and women between 18 and 65 years of age from Mexico’s urban areas, with children between 1 and 12 years of age, with interests in parenting, paternity, maternity, and fitness. In total, 1210 caregivers provided answers to the scale via a digital form, but we eliminated data from 25 caregivers with children whose ages fell outside of the age range. The final sample was subdivided into two random subsamples for the EFA (n = 581) and CFA (n = 604).

#### 2.3.2. Materials and Instruments

Each participating caregiver provided their digital informed consent. The scale, which comprised 80 items regarding the frequency of parental behaviors exhibited while feeding a child in the last week, was distributed via Google Forms. The participants’ response options were never (1), a few times (2), sometimes (3), many times (4), and always (5).

#### 2.3.3. Data Analysis

Skewness and kurtosis values were used to analyze the normality assumption for each item’s distribution. We excluded items with high skewness values and kurtosis > |1.5|. We confirmed the adequacy of the sampling using the Kaiser–Meyer–Olkin (KMO) measure (≥0.6), and the factorability of the data was confirmed using Bartlett’s test of sphericity (*p* < 0.05). We performed an exploratory factor analysis (EFA), according to recommendations [18,19], using the maximum likelihood extraction method and Kaiser’s Oblimin rotation to study the scale’s psychometric properties via SPSS v. 29. Modeling was carried out by eliminating the items with a factorial weight of less than 0.350 or those with a weight greater than 0.300 for more than one factor. A confirmatory factorial analysis (CFA) was then performed using AMOS v.29. For reliability, Cronbach’s alpha value was calculated for the final version of the scale (40 items), and McDonald’s omega value was calculated for each dimension.

## 3. Results

### 3.1. Participants

The caregivers were 32.7 (7.6) years old, with a mean BMI of 26.9 (9.3). Among the participants, 52.7% reported answering the scale while thinking about their behaviors when feeding a girl, while the rest did so while thinking about feeding a boy. The child’s age was 4.8 (3) years. According to the regionalization reported in the Encuesta Nacional de Salud y Nutrición, 35.4% of participants lived in the urban areas of the western region, 30.9% lived in the northern part, 24.5% lived in the central region, and 9.2% lived in the southern region [20]. Finally, 97% of the participants were women, 2.4% were men, and 0.6% preferred not to indicate their gender; 89.9% were mothers, 2.6% were fathers, 2.5% were aunts, 1.9% were grandparents, 0.6% were cousins, and 2.2% were unrelated to the child. Table 2 presents the economic income ranges and academic levels of the participants.

### 3.2. Exploratory Factorial Analysis

The KMO (0.892) value and Bartlett’s test of sphericity (X2 = 13,141.025, df = 1176, *p =* 0.000) indicated an adequate sample and the utility of the factorial analysis. The items were grouped into 11 factors in the EFA, which explained 63.9% of the accumulated variance.

As shown in Table 3, the eleven factors obtained from the rotation, given its coincidence with the theoretical construct, were named as follows: (1) disposition of non-recommended foods (DNR), (2) nutritional education (EN), (3) pressure to eat (P), (4) praise for healthy eating (El), (5) meal times (MT), (6) monitoring of consumption (Mn), (7) structured offer of fruits and vegetables (OFV), (8) consumption conditioning (Co), (9) overt restriction (RO), (10) guided choices (EG), and (11) covert restriction (RC). This version of the instrument comprised 49 items.

### 3.3. Confirmatory Factor Analysis

Different models were used for the CFA, as indicated in Table 4. The proposal with ten factors and 40 items showed the best properties and fit (CMIN = 1531.5, *p* < 0.001, CMIN/df = 2.20, RSEA = 0.045, CFI = 0.92, TLI, 0.91, and NFI = 0.87). Among the eleven items that showed a weight of less than 0.490 were those belonging to the meal time (MT) factor, so this factor was eliminated in the final version of the instrument.

**Figure 2:**
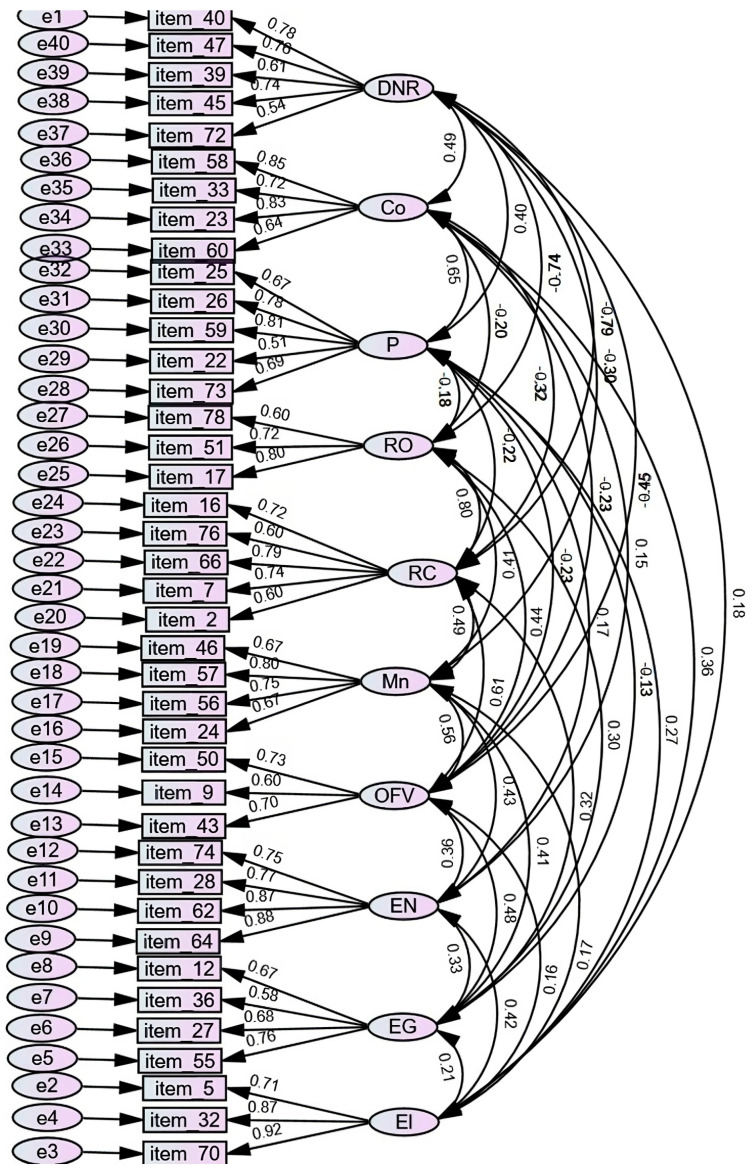
Confirmatory factor analysis diagram of the final model with ten factors and 40 items. Note: DNR = disposition of non-recommended foods; RO = overt restriction; RC =covert restriction; P = pressure to eat; Co = consumption conditioning; Mn = monitoring; OFV = structured offer of fruits and vegetables; EN = nutritional education; EG = guided choices; El = praise for healthy eating.

Regarding the correlations between the ten factors, as shown in Figure 2, correlations were high among the factors of coercive control, specifically the correlation of the practice disposition of non-recommended foods (DNR) with overt (RO, −0.736) and covert restriction (RC, −0.791), as well as pressure to eat (P, 0.402) and the consumption conditioning (Co, 0.49). P correlated with Co (0.65). Factors of RC were correlated with RO (0.795) and negatively correlated with Co (−0.32). There were also correlations between the positive practices of autonomy or structure, specifically monitoring (Mn) with the structured offer of fruits and vegetables (OFV, 0.56), as well as with nutritional education (EN, 0.43) and guided choices (EG, 0.41). The EG correlated with OFV (0.48) and EN (0.33). The EN correlated with OFV (0.36) and praise behaviors (El, 0.41). In addition, positive correlations were observed between the practice of CR with OFV (0.61), with Mn (0.49), and with EG (0.32). The behaviors of RA correlated with Mn (0.41), OFV (0.44), and with EG (0.3). The practice of Co correlated with El (0.36). The practice of DNR was negatively correlated with Mn (−0.31), with OFV (−0.45), and with CR (−0.79).

### 3.4. Consistency Analysis

The final version with 40 items obtained a Cronbach’s alpha value of 0.816, and a single-item consistency analysis showed a Cronbach’s alpha greater than 0.8 in all cases (Table 5), indicating a reliable instrument. In addition, McDonald’s omega was acceptable for each dimension (>0.7) (see Table 3).

## 4. Discussion

The present work provides evidence of the construct validity and internal consistency of the self-applicable Scale on Parental Feeding Behaviors (ECOPAL). For this reason, it is considered suitable for evaluating the frequency with which caregivers or adults responsible for feeding children between 1 and 12 years of age perform positive and negative parental eating behaviors to promote healthy eating. The scale’s internal consistency was analyzed, and evidence of its construct validity was provided. Similarly, the scale provides empirical evidence of the theoretical basis [6] with respect to the classification of parental feeding practices. The results were compared with the results of two other instruments with the same theoretical basis, the HomeSTEAD family food practices survey [10,15], which does not have a Spanish version, and the Comprehensive Feeding Practices Questionnaire in Spanish to Mexican Mothers [14], which changes the original construct of parental feeding practices to include attitudes and beliefs towards food.

The dimensions of the ECOPAL scale showed adequate consistency (>0.68); some were better than the original version of the HomeSTEAD survey (>0.62) and its Portuguese version (>0.61). Although the ECOPAL questionnaire evaluates fewer practices than the three subscales of the HomeSTEAD survey, it can determine them globally and comprehensively on the same scale. It can account for the frequency of behaviors of each practice and provide information regarding their relative frequency to other practices.

Compared with the Comprehensive Feeding Practices Questionnaire for Mexican Mothers (CIPA) [14], ECOPAL presented better consistency of global scale (α = 0.64 vs. α = 0.816, respectively). The CIPA remained the modeling’s dimension with high consistency (α = 0.965) but with items that evaluate beliefs on modeling more than actions.

The instrument resulting from the psychometric validation process shows adequate construct validity, since it presents nine of the thirteen theoretical constructs, namely restriction (overt and covert); pressure to eat; the consumption conditioning; food disposal (not recommended and fruits and vegetables); guided choices; unstructured practices (meal times); intake monitoring; nutritional education; and motivation for healthy eating (praise). In addition, some components were grouped by the type of food they offer, such as the structured offer of fruits and vegetables (OFV) and the disposition of non-recommended foods (DNR). This finding is particularly relevant because it accounts for the practice of food availability and is part of the structure construct [5].

The MT factor, which refers to a lack of structure at meal times, is obtained via the EFA. These items can account for unstructured eating practices. However, the MT factor was not maintained in the CFA; it is considered theoretically relevant, since meal times and accompanying the child and sharing food are indicated as part of the modeling climate for children’s eating behaviors [21].

The constructs not maintained in the instrument from the EFA were modeling, eating habits, the involvement of the child in eating, and negotiation. It should be noted that these practices belong to categories of structure and autonomy support. Five items that presented significant biases were lost from the beginning, as evidenced by high kurtosis and asymmetry values (>1.5); these were structure practices such as modeling and eating habits. The values could have occurred since caregivers reported having performed these behaviors with extreme scores, such as never or always. The negotiation construct disappeared due to the low communality (<0.3) presented by the items from the extraction method and was affected by having only included three items in this factor since its creation. Reviewing the wording and including more items in future applications to evaluate this parental practice would be convenient. Although a minimum of three or four items per factor is recommended [22], if there is a minimum of 200 cases, which this study fulfills, having 581 cases for the EFA, current recommendations indicate that the more items there are that accurately measure a factor, the more determined this factor will be and the more stable the factorial solution will be [19]. Finally, the items regarding the child’s involvement in feeding were lost for two reasons: two items had saturations lower than 0.3, and the rest presented saturations > 0.3 with more than one factor, which was shared with the nutritional education factor and with consumption motivation. After considering the reviewed literature regarding recommendations for conducting an exploratory factor analysis [19,22], these items were eliminated, and a detailed assessment is required to determine whether their modification is recommended for their inclusion in a new version of the scale [19], or if it is necessary to add new items with similar content to adequately sample the content of the factor [18].

Of the factors resulting from the EFA and CFA, four of these report parental feeding practices associated with the formation of unhealthy eating habits; these are the disposal of non-recommended foods (DNR), the pressure to eat (P), consumption conditioning (Co), and overt restriction (RO), which are considered coercive control practices. On the other hand, the factors associated with the promotion of healthy eating habits are those practices that support autonomy, in this case, nutritional education (EN), praise for healthy eating (El), guided choices (EG), and those relating to structure, namely the structured offer of fruits and vegetables (OFV), monitoring of consumption(Mn), and covert restriction (CR). This classification is based on the reviewed literature [5,9,21] and is supported by the correlations found in the CFA.

The correlations shown by the CFA strengthen the categorization of the practices of open or manifest restriction, the conditioning of consumption, and the pressure to eat as coercive control, since they had positive and high correlations between them but negative correlations with other practices, while the practices of monitoring, the structured offer of fruits and vegetables, nutritional education, praising healthy eating, and guided choices had positive correlations between them and negative correlations with the practices of coercive control, thus strengthening their categorization as practices of autonomy support and structure that, unlike the coercive practices, have been associated in the literature with the formation of healthy eating habits. The disposition of non-recommended foods practice had negative correlations with the structure practices; likewise,: monitoring and the offering of fruits and vegetables had correlations with the practices of overt and covert restriction, but did not correlate with behaviors supporting autonomy, inferring the confusing role that caregivers bringing these foods home can play in promoting healthy eating in children. Regarding covert restriction, positive correlations were obtained with structure practices, such as monitoring and offering fruits and vegetables, and with autonomy support practices, such as guided choices, in addition to a negative correlation with the practice of the disposition of non-recommended food. The preceding result could suggest that this practice, when related to the limited availability of foods not recommended for daily consumption, is considered a practice of structure rather than coercive control, an aspect already discussed in the literature [5,9].

The limitations of this study are the same as those linked to the self-report questionnaires, such as the effects of memory bias or social desire [23], which were taken care of with a frequency scale made explicit in the instructions to consider the events of the last week. Regarding social desirability, care was taken to present the items randomly and to use some reversible ones. However, in the factorial analysis, some of these elements were eliminated. Some authors recently indicated that inverse items could contribute more to confusion than to verifying answers, evidencing that these items required adjustments to the method [24].

Some limitations of this study may be related to the process of selecting samples from social networks, because we considered filters such as having an interest in paternity issues, which represents a bias in the representation of Mexican caregivers, since there may be a significant number of them who do not search for parenting issues on social networks; this might explain the high academic level or low economic level of the sample, which are other possible biases.

Another limitation of this study was the loss of items that evaluated modeling, which is a practice of great interest; a review of the wording of these items is necessary to reduce the response bias of never or always, and conducting a future factorial analysis with them is necessary to be able to include the evaluation of this practice.

Finally, the application of this scale occurred online. Although it is undeniable that this has advantages, such as the possibility of reaching a global population, achieving very large sample sizes, the flexibility of the survey design, the speed and timeliness of administration, and ability to force response completion [25], it is also true that it presents drawbacks. Some prominent factors are the inability to provide clear, one-on-one instructions to respondents, inherent sampling biases, a self-selection bias, variability among respondents in their ability to access the survey due to device limitations, and connectivity issues, among others (for reviews, see [25]).

## 5. Conclusions

Based on the results previously described and discussed, it is concluded that the instrument presented herein, the Scale on Parental Feeding Behavior (ECOPAL), which contains 40 items in its final version, has adequate internal consistency indexes and shows evidence of construct validity. We consider it adequate and useful for evaluating the frequency of parental eating behaviors, both positive and negative, which factor into the formation of healthy eating habits in children. The above is very useful when planning interventions at both the primary and secondary levels to prevent health problems related to poor nutrition, such as childhood obesity, as well as in the prevention of eating disorders, and more specifically by guiding caregivers on how to deal with or prevent problems with feeding behavior, such as food selectivity, neophobia, emotional eating, or over-eating, more effectively.

## Figures and Tables

**Table 1:** The theoretical basis of the instrument.

Category	Construct	Items
Coercive control: parental pressure and the domination of the child’s feelings, thoughts, and behaviors.	Restriction: the caregiver places strict limitations on access to food, usually to control the consumption of unhealthy foods.	7, 16, 17, 40, 51, 66, 71, 78, 80
	Pressure to eat: the caregiver insists, verbally or through physical force, that the child consume more food.	6, 23, 25, 26, 35, 59, 73
	Determinants of consumption: the caregiver offers food that the child likes to encourage consumption or promises to carry out an activity in exchange for consuming a specific food.	13, 21, 33, 58, 69, 77
Structure in feeding: how the parents organize the environment to facilitate the development of skills in the child.	Food availability: the variety of foods the caregiver offers and has available for the child’s consumption.	2, 15, 38, 42, 45, 47, 50, 54, 67, 69, 76, 79
	Modeling: the caregiver purposely shows their eating habits to encourage similar behaviors in the child or involuntarily displays unhealthy food consumption in front of the child.	8, 18, 19, 30, 37, 61, 65, 72
	Eating habits: the caregiver creates a consistent routine that is implemented during feeding time, considering the location, time, and presence of family members.	3, 9, 14, 39, 43, 44, 75
	Unstructured practices: the caregiver allows the child to have complete control during the feeding period, including the timing, frequency, and type of food.	10, 20, 34, 48, 52
	Guided choices: the caregiver allows the child to choose what to consume among options proposed by the caregiver.	12, 27, 36, 55
	Monitoring feeding: the caregiver actively tracks what and how much the child is eating, ensuring that the child eats enough healthy food and avoids unhealthy food.	22, 24, 46, 56, 57
Support and autonomy: allowing the child, according to their age, to make choices, allowing them to self-regulate in the future without their parents.	Motivation for food consumption and interaction: the caregiver praises the child for consuming new or healthy food.	5, 11, 32, 70
	Nutritional education and reasoning: the caregiver explains the nutritional qualities of food, the benefits of eating healthy foods, and the consequences of eating unhealthy foods.	28, 62, 64, 74
	The involvement of the child in eating: the caregiver actively includes the child in the choice of food when buying and preparing food, allows them to interact with the food, and is interested in knowing the child’s perception of the food. Food for consumption.	1, 31, 49, 53, 63, 68
	Negotiation: the caregiver engages the child in reaching an acceptable compromise about what and how much the child will eat.	4, 29, 41

Note: operational definitions were based on Vaughn and colleagues, 2016 [5] and translated into Spanish in Mexico in 2018 [6].

**Table 2:** Economic income ranges and academic levels of the participants.

	Elementary *f*	High School *f*	College *f*	Postgraduate *f*
MXN 4999 or less	36 (36%)	49 (17%)	29 (5%)	5 (3%)
MXN 5000 to MXN 9999	35 (35%)	109 (37%)	158 (27%)	10 (5%)
MXN 10,000 to MXN 14,999	10 (10%)	64 (22%)	132 (22%)	32 (16%)
MXN 15,000 to MXN 19,999	3 (3%)	29 (10%)	77 (13%)	26 (13%)
MXN 20,000 to MXN 24,999	2 (2%)	11 (4%)	55 (9%)	34 (17%)
MXN 25,000 to MXN 29,999	2 (2%)	2 (1%)	31 (5%)	29 (15%)
MXN 30,000 or more	1 (1%)	7 (7%)	42 (22%)	43 (22%)
I prefer not to answer	10 (10%)	24 (8%)	68 (11%)	20 (10%)

Note: Incomes are provided by month in Mexican pesos. n = 1185, *f* = Frecuencia.

**Table 3:** Factorial rotation matrix of the EFA items and internal consistency with Cronbach’s alpha and McDonald’s omega.

Factor Names and Items in Spanish and English	DNR	EN	P	El	MT	Mn	OFV	Co	RO	EG	RC
**Disposición de alimentos no recomendables (DNR).** ***Disposition of non-recommended foods.***	α = 0.847	**ω = 0.845**									
40. Ofrezco al niño(a), botanas, dulces, postres o cereales endulzados (por ejemplo: Zucaritas, Froot Loops, papas fritas, dulces, frituras, caramelos, chocolates, galletas, pastelitos, etc.). *I offer the child snacks, sweets, desserts, or sweetened cereals.*	−0.764										
47. Ofrezco al niño(a) alimentos procesados y comida rápida (por ejemplo: alimentos enlatados, embutidos como salchicha o jamón, hamburguesas, pizza, pollo frito, etc.). *I offer the child processed foods and fast food.*	−0.661										
39. Le doy al niño(a) botanas, dulces o postres a media mañana y media tarde. *I give the child snacks, sweets, or desserts in the mid-morning and mid-afternoon.*	−0.575										
45. Ofrezco al niño(a) bebidas endulzadas procesadas (por ejemplo: jugos procesados, refrescos, bebidas lácteas endulzadas como yogurt, Danonino, Yakult, etc.). *I offer the child processed sweetened beverages.*	−0.523										
72. Muestro mi agrado al comer botanas, dulces, postres o cereales endulzados, antojitos mexicanos o alimentos procesados frente al niño(a). *I show my pleasure by eating snacks, sweets, sweetened desserts or cereals, Mexican fried food, or processed foods in front of the child*.	−0.482										
* 37. Consumo frente al niño(a) botanas, dulces o postres, antojitos mexicanos o comida procesada (por ejemplo: papitas, dulces, chocolates, galletas, pastelitos, sopes, etc.). *I eat snacks, sweets or desserts, Mexican fried foods, or processed food in front of the child.*	−0.449										
**Educación Nutricional (EN).** ***Nutritional education.***		α = 0.856						ω = 0.869			
64. Cuando el niño(a) rechaza un alimento le digo los beneficios de consumirlo. *When the child rejects a food, I tell him/her the benefits of consuming it.*		0.822									
62. Le digo al niño(a) de los beneficios de consumir alimentos como frutas y verduras de forma cotidiana. *I tell the child about the benefits of consuming foods such as fruits and vegetables daily*		0.802									
28. Le digo al niño(a) las propiedades nutricionales de los alimentos que le ofrezco. *I tell the child about the nutritional properties of the food that I offer*		0.696									
74. Le digo al niño(a) de los efectos que tienen alimentos como botanas, dulces o postres si se consumieran de forma cotidiana. *I tell the child about the effects of foods such as snacks, sweets or desserts if consumed daily.*		0.663									
* 63. Involucro al niño(a) en la preparación de los alimentos. *I involve the child in the preparation of food.*		0.413									
**Presión para comer (P).** ***Pressure to eat.***	α = 0.818					**ω = 0.828**					
25. Durante la comida le insisto al niño(a) para que siga comiendo a pesar de que diga que ya está lleno. *During the meal, I insist that the child continue to eat even if he/she says he/she is full.*			0.745								
59. Le insisto al niño(a) para que se coma todo lo que le sirvo en cada comida. *I insist that the child eat everything I serve him/her at each meal.*			0.674								
26. Le insisto al niño(a) que se termine un alimento o porción específica de lo que le sirvo en cada comida. *I urge the child to finish a specific food or portion that I serve at each meal.*			0.667								
* 35. Insisto al niño(a) a que siga comiendo introduciendo alimento en su boca. *I urge the child to continue eating by introducing food into his/her mouth.*			0.554								
22. Permito que el niño(a) deje de comer cuando dice que ya está lleno (R). *I allow the child to stop eating when he/she says he/she is full (R).*			0.463								
73. Le digo al niño(a) que la comida no se desperdicia por lo que se la tiene que acabar. *I tell the child that food is not wasted, so he/she must finish it.*			0.388								
**Elogios ante alimentación saludable (El).** ***Praise for healthy eating.***	α = 0.857			ω = 0.861							
70. Felicito o elogio al niño(a) cuando prueba un nuevo alimento. *I congratulate or praise the child when trying a new food.*				0.927							
32. Felicito o elogio al niño(a) por haber probado un alimento que había rechazado. *I congratulate or praise the child for tasting a food that he/she had rejected.*				0.817							
5. Felicito o elogio al niño(a) cuando come alimentos como frutas o verduras. *I congratulate or praise the child when he/she eats fruits or vegetables.*				0.642							
**Horarios de comida desestructurados (MT).** ***Meal times.***	α = 0.684			ω = 0.746							
* 34. El niño(a) no tiene horario de comida y puede comer cuando quiera. *The child does not have a meal schedule, he/she can eat whenever he/she wants.*					0.858						
* 44. El niño(a) tiene horarios establecidos para cada una de las comidas del día(R). *The child has established times for each of the meals of the day(R)*					0.566						
* 20. Permito que el niño(a) decida cuándo quiere comer. *I allow the child to decide when he/she wants to eat.*					0.545						
**Monitoreo del consumo (Mn).** ***Monitoring of consumption***	α = 0.795			ω = 0.787							
56. Llevo la cuenta de las botanas, dulces y postres que consume el niño(a) durante el día (por ejemplo: Zucaritas, Froot Loops, papas fritas, dulces, frituras, caramelos, chocolates, galletas, pastelitos, etc.) *I keep track of the snacks, sweets and desserts that the child consumes during the day.*						0.841					
24. Llevo la cuenta de las bebidas endulzadas que toma el niño(a) durante el día (por ejemplo: jugos de fruta natural, jugos procesados, aguas frescas, refrescos, bebidas lácteas endulzadas como yogurt, Danonino, Yakult, etc.) *I keep track of the sweetened drinks the child drinks during the day*						0.686					
57. Llevo la cuenta de las frutas y verduras que come el niño(a) durante el día. *I keep track of the fruits and vegetables that the child eats during the day*						0.552					
46. Llevo la cuenta de la cantidad de agua natural que bebe el niño(a) durante el día. *I keep track of the amount of natural water the child drinks during the day*						0.393					
**Oferta estructurada de Frutas y Verduras (OFV).** ***Structured offer of fruits and vegetables***	α = 0.729				ω = 0.733						
43. Le doy al niño(a) frutas o verduras a media mañana y media tarde. *I give the child fruits or vegetables in the mid-morning and mid-afternoon*							0.837				
9. Le doy al niño(a) algún alimento de colación a media mañana y media tarde. *I give the child some snack food in the mid-morning and mid-afternoon*							0.658				
50. Ofrezco al niño(a) diferentes frutas o verduras en cada comida. *I offer the child different fruits or vegetables at each meal.*							0.513				
**Restricción abierta o manifiesta (RO).** ***Overt restriction.***	α = 0.785					ω = 0.786					
78. Le niego el consumo de comidas preparadas con aceite como antojitos mexicanos (por ejemplo: sopes, enchiladas, flautas, etc.). *I deny the intake of foods prepared with oil, such as Mexican fried food.*								0.870			
* 30. Evito tener al alcance del niño(a) antojitos mexicanos (por ejemplo: sopes, enchiladas, flautas, etc.). *I avoid having Mexican fried food within the reach of the child.*								0.605			
* 54. Ofrezco al niño(a) antojitos mexicanos (por ejemplo: sopes, enchiladas, flautas, etc.)(R) *I offer the child Mexican fried food.*								0.572			
51. Le niego el consumo de botanas, dulces, postres o cereales endulzados al niño(a) (por ejemplo: Zucaritas, Choco Krispis, papas fritas, frituras, dulces, chocolates, galletas, pastelitos, etc.). *I deny the intake of snacks, sweets, desserts or sweetened cereals to the child.*								0.417			
17. Le niego el consumo de alimentos procesados y comida rápida (por ejemplo: alimentos enlatados, embutidos como salchicha o jamón, hamburguesas, etc.). *I deny the intake of processed foods and fast food to the child.*								0.406			
**Condicionamiento del consumo de alimentos (Co).** ***Consumption conditioning.***	α = 0.828					ω = 0.831					
58. Cuando el niño(a) rechaza un alimento le digo que realizará alguna actividad a cambio de terminarse el alimento (por ejemplo: ver televisión, salir al parque, jugar videojuegos). *When the child rejects a food, I tell him/her that he/she will do some activity in exchange for finishing the food*.									0.725		
23. Le digo al niño(a) que termine todo lo que está en su plato a cambio de permitirle hacer cosas que le gustan (por ejemplo: ver TV, salir a jugar, etc.). *I tell the child to finish everything on his plate in exchange for allowing him to do things he likes.*									0.694		
33. Le digo al niño(a) que si se porta bien le daré un alimento de su agrado. *I tell the child that if he behaves well I will give him/her a food he likes.*									0.680		
* 13. Le ofrezco al niño(a) algún alimento de su agrado para celebrar algo que logró con éxito. *I offer the child some food they like to celebrate something they have successfully achieved.*									0.513		
60. Cuando el niño(a) rechaza un alimento le ofrezco otro de su agrado con la condición de que consuma el que rechazó. *When the child rejects a food, I offer him/her another to his/her liking on the condition that he eats the one he refused.*									0.432		
**Elecciones Guiadas (EG).** ***Guided choices.***	**α = 0.742**					**ω = 0.743**					
55. Le sirvo al niño(a) dos o más opciones del mismo grupo de alimentos para que al menos coma uno de ellos. *I serve the child two or more options from the same food group so that they eat at least one of them.*										0.732	
36. Cuando el niño(a) rechaza un alimento le ofrezco otro del mismo grupo de alimentos. *When the child rejects a food, I offer another from the same food group.*										0.554	
27. Pongo al centro de la mesa dos o más opciones del mismo grupo de alimentos para que el niño(a) coma lo que prefiera. *I put two or more options from the same food group in the center of the table so that the child can eat what he/she prefers*.										0.554	
12. Le muestro al niño(a) dos o más opciones del mismo grupo de alimento y le pregunto cuál prefiere comer. *I show the child two or more options from the same food group and ask him/her which he/she prefers to eat,*										0.504	
**Restricción encubierta (RC).** ***Covert restriction.***	α = 0.829				ω = 0.831						
2. Evito llevar a casa bebidas que considero no recomendables para consumo diario. *I avoid taking home drinks that I consider not recommended for daily consumption.*											0.552
16. Evito tener al alcance del niño(a) botanas, dulces, postres o cereales endulzados (por ejemplo, papitas, frituras, dulces, chocolates, galletas, pastelitos, etc.). *I avoid having snacks, sweets, desserts, or sweetened cereals within the child’s reach.*											0.567
7. Evito tener al alcance del niño(a) alimentos procesados y comida rápida (por ejemplo: alimentos enlatados, embutidos como salchicha o jamón, hamburguesas, etc.). *I avoid having processed foods and fast food within the child’s reach.*											0.500
66. Evito tener al alcance del niño(a) bebidas endulzadas procesadas (por ejemplo: jugos y néctares procesados, refrescos, bebidas lácteas endulzadas como yogurt, Danonino, Yakult, etc.). *I avoid having processed sweetened beverages within the child’s reach.*											0.472
76. Evito llevar a casa alimentos que considero no recomendables para el consumo diario. *I avoid taking home foods that I consider not recommended for daily consumption*											0.432
Global	α = 0.795										

Note: The extraction method was the maximum likelihood. The rotation method was Oblimin with Kaiser normalization. The factors are the result of the EFA. Items with * were eliminated for the final version of the instrument according to the confirmatory factor analysis, thus revealing better adjustment and better psychometric properties. The reliability of the total scale with 40 items was α = 0.816.

**Table 4:** Comparison of values of each confirmatory factor analysis model.

Models	Chi-Square	*p*	Chi- Normed Square	Root Mean Square Error of Approximation (RMSEA)	Comparative Fit Index (CFI)	Non-Normalized Index of Fit (NNFI o TLI)	Normalized Fit Index (NFI)	Parsimonious Comparative Fit (PCFI)	Parsimonious Normed Fit (PNFI)	Akaike’s Information Criterion (AIC)
Expected		>0.05	<3/5	<0.05/0.08	0.9–1	0.9–1	0.9–1	0.9–1	Close to 1	Close to 0
11 factors and 49 items	2672.22	0	2.49	0.05	0.871	0.862	0.815	0.769	0.713	2897.89
10 factors and 40 items	1531.5	0	2.204	0.045	0.924	0.915	0.871	0.824	0.776	1861.53

**Table 5:** Consistency analysis, subtracting the unique elements.

	Mean Scale If the Element Has Been Suppressed	Scale Variance If the Element Has Been Suppressed	Total Item Correlation Corrected	Cronbach’s Alpha If the Item Has Been Deleted
item_76	114.5620	257.111	0.294	0.812
item_66	114.6616	257.923	0.214	0.815
item_16	114.7806	258.856	0.198	0.815
item_7	114.8633	257.064	0.249	0.814
item_2	114.7865	256.700	0.255	0.813
item_27	115.9544	256.564	0.277	0.813
item_12	115.4869	253.568	0.340	0.811
item_36	115.6481	255.541	0.309	0.812
item_55	115.6422	255.103	0.352	0.811
item_60	116.2844	255.580	0.329	0.811
item_23	116.1688	255.830	0.272	0.813
item_33	116.1426	257.658	0.227	0.814
item_58	116.3131	254.046	0.341	0.811
item_51	115.2641	258.632	0.199	0.815
item_17	115.2751	257.642	0.246	0.814
item_50	114.5308	254.835	0.351	0.811
item_78	115.5249	258.905	0.184	0.816
item_9	114.6101	257.103	0.265	0.813
item_43	114.4464	256.806	0.308	0.812
item_46	115.1139	247.142	0.406	0.808
item_57	114.9468	245.550	0.469	0.806
item_56	114.7511	245.739	0.431	0.807
item_24	114.7013	249.301	0.333	0.811
item_32	114.5316	248.908	0.424	0.808
item_5	114.6034	249.701	0.375	0.809
item_70	114.5080	246.946	0.470	0.806
item_73	115.9004	255.007	0.261	0.813
item_22	116.5595	263.691	0.093	0.817
item_25	116.5063	259.929	0.216	0.814
item_26	115.7418	254.661	0.299	0.812
item_59	115.7932	253.549	0.315	0.812
item_74	114.7806	245.759	0.470	0.806
item_28	115.3823	243.539	0.508	0.804
item_62	114.4481	245.822	0.486	0.806
item_64	115.0093	241.947	0.574	0.802
item_72	115.6810	269.412	−0.089	0.822
item_45	116.0700	270.036	−0.113	0.822
item_39	116.2692	267.771	−0.036	0.820
item_47	116.0219	270.445	−0.135	0.822
item_40	115.9443	268.786	−0.071	0.821

Note: the global Cronbach’s alpha value is 0.816.

## Data Availability

Data are available upon request due to privacy restrictions. The data presented in this study are available upon request from the corresponding author.

## References

[1] Fondo de las Naciones Unidas para la Infancia (UNICEF) (2019). Estado Mundial de la Infancia 2019—Niños, Alimentos y Nutrición.

[2] Fondo de las Naciones Unidas para la Infancia (UNICEF) (2018). Salud y Nutrición.

[3] Padilla-Vinueza V.E.P., Tisalema H., Gavilanez R.I.A., Cunalata E.I.J., Carrión A.A.M., Aguilar A.D.S. (2022). Obesidad infantil y métodos de intervención. Dominio Cienc..

[4] Martínez-Munguía C., Navarro-Contreras G. (2014). Factores Psicológicos, Sociales y Culturales del Sobrepeso y la Obesidad Infantil y Juvenil en México. Rev. Médica Inst. Mex. Seguro Soc..

[5] Vaughn A.E., Ward D.S., Fisher J.O., Faith M.S., Hughes S.O., Kremers S.P., Musher-Eizenman D.R., O’connor T.M., Patrick H., Power T.G. (2016). Fundamental constructs in food parenting practices: A content map to guide future research. Nutr. Rev..

[6] González-Torres M.L., Esqueda C.N., Vacio M.A. (2018). Prácticas alimentarias parentales y su relación con la conducta alimentaria infantil: Problemas para la explicación. Rev. Mex. Trastor. Aliment..

[7] Shloim N., Edelson L.R., Martin N., Hetherington M.M. (2015). Parenting Styles, Feeding Styles, Feeding Practices, and Weight Status in 4–12 Year-Old Children: A Systematic Review of the Literature. Front. Psychol..

[8] Castrillón I.C., Giraldo O. (2014). Prácticas de alimentación de los padres y conductas alimentarias en niños: ¿existe información suficiente para el abordaje de los problemas de alimentación?. Rev. Psicol. Univ. Antioq..

[9] González-Torres M.L., González L.A., Altamirano A., Pedroza F., Díaz R., Reyes L.I., López F. (2018). Interacciones cuidador-niño durante la alimentación y el aprendizaje temprano de hábitos alimentarios. Aportaciones Actuales a la Psicología Social (IV).

[10] Vaughn A.E., Dearth-Wesley T., Tabak R.G., Bryant M., Ward D.S. (2017). Development of a comprehensive assessment of food parenting practices: The home self-administered tool for environmental assessment of activity and diet family food practices survey. J. Acad. Nutr. Diet..

[11] Birch L.L., Fisher J.O., Grimm-Thomas K., Markey C.N., Sawyer R. (2001). Confirmatory factor analysis of the Child Feeding Questionnaire: A measure of parental attitudes, beliefs, and practices about child feeding and obesity proneness. Appetite.

[12] Navarro-Contreras G., Lagunes I.R. (2016). Validación Psicométrica de la Adaptación Mexicana del Child Feeding Questionnaire. Acta Investig. Psicol..

[13] Musher-Eizenman D., Holub S. (2007). Comprehensive Feeding Practices Questionnaire: Validation of a New Measure of Parental Feeding Practices. J. Pediatr. Psychol..

[14] Ángel J.G., Flores Y.P., Trujillo P.E.H., Ávila H.A., Gutiérrez J.M.V. (2021). Confirmatory factor analysis of the Comprehensive Feeding Practices Questionnaire in Mexican mothers of preschool children. Nutr. Hosp..

[15] Afonso L., Castro J., Parente N., Torres S. (2020). A Comprehensive Assessment of Food Parenting Practices: Psychometric Properties of the Portuguese Version of the HomeSTEAD Family Food Practices Survey and Associations with Children’s Weight and Food Intake. Eur. J. Investig. Health Psychol. Educ..

[16] Escobar-Pérez J., Cuervo-Martínez Á. (2008). Validez de Contenido y Juicio de Expertos: Una Aproximación a su Utilización. Av. Medición.

[17] Van Widenfelt B.M., Treffers P.D.A., de Beurs E., Siebelink B.M., Koudijs E. (2005). Translation and Cross-Cultural Adaptation of Assessment Instruments Used in Psychological Research with Children and Families. Clin. Child. Fam. Psychol. Rev..

[18] Costello A.B., Osborne J. (2005). Best practices in exploratory factor analysis: Four recommendations for getting the most from your analysis. Pract. Assess. Res. Eval..

[19] Lloret-Segura S., Ferreres-Traver A., Hernández-Baeza A., Tomás-Marco I. (2014). El análisis factorial exploratorio de los ítems: Una guía práctica, revisada y actualizada. An. Psicol. Ann. Psychol..

[20] Shamah-Levy T., Vielma-Orozco E., Heredia-Hernández O., Romero-Martínez M., Mojica-Cuevas J., Cuevas-Nasu L., Santaella-Castell J.A., Rivera-Dommarco J. (2020). Encuesta Nacional de Salud y Nutrición [ENSANUT] 2018–19: Resultados Nacionales.

[21] Jimeno-Martínez A., Maneschy I., Rupérez A.I., Moreno L.A. (2021). Factores Determinantes del Comportamiento Alimentario y su Impacto Sobre la Ingesta y la Obesidad en Niños. J. Behav. Feed..

[22] Ferrando P.J., Anguiano-Carrasco C. (2010). El Análisis Factorial Como Técnica de Investigación en Psicología. Papeles Psicól..

[23] Del Valle M., Zamora E.V. (2021). El uso de las Medidas de Auto-Informe: Ventajas y Limitaciones en la Investigación en Psicología. Altern. Psicol..

[24] Tomás J.M., Requena P.S., Germes A.O., Llinares L.G., Moral J.C.M. (2012). Efectos de Método Asociados a Ítems Invertidos vs. Ítems en Negativo. Rev. Mex. Psicol..

[25] Jaeger S.R., Cardello A.V. (2022). Factors affecting data quality of online questionnaires: Issues and metrics for sensory and consumer research. Food Qual. Prefer..

